# An Australian housing conditions data infrastructure

**DOI:** 10.1038/s41597-023-02739-2

**Published:** 2023-11-21

**Authors:** Emma Baker, Claire Morey, Lyrian Daniel, Andrew Beer, Rebecca Bentley, Wendy Stone, Steven Rowley, Christian A. Nygaard, Kerry London

**Affiliations:** 1 Australian Centre for Housing Research, The University of Adelaide, Adelaide, 5005 Australia; 2 UniSA Creative, The University of South Australia, Adelaide, 5000 Australia; 3 UniSA Business, The University of South Australia, Adelaide, 5000 Australia; 4 Melbourne School of Population and Global Health, The University of Melbourne, Melbourne, 3000 Australia; 5 Centre for Urban Transitions, Swinburne University of Technology, Melbourne, 3122 Australia; 6 School of Accounting, Economics and Finance, Curtin University, Perth, 6102 Australia; 7 Office of the Pro Vice Chancellor Research, Torrens University Australia, Sydney, 2007 Australia

**Keywords:** Geography, Social sciences

## Abstract

For the past two decades, researchers and policy makers have known very little about conditions within Australia’s housing stock due to a lack of systematic and reliable data. In 2022, a collaboration of Australian universities and researchers commissioned a large survey of 22,550 private rental, social rental and homeowner households to build a data infrastructure on the household and demographic characteristics, housing quality and conditions in the Australian housing stock. This is the third and largest instalment in a national series of housing conditions data infrastructures.

## Background & Summary

For all Australians, either via renting or owning, housing offers much more than just shelter^1^. It provides space for raising families, for leisure and rest, and increasingly, it doubles as a workspace, with a reported 20 per cent (2.5 million) of employed Australians working from home on the Census Day in 2021^2^. Housing also impacts our mental and physical health due to factors including cold, mould, poorly managed maintenance issues, unaffordability, and inequality^3^.

Despite the centrality of housing in our everyday lives, the research and policy community has limited large-scale data available to describe Australian housing conditions and the corresponding experience and aspirations of householders. Almost all other developed nations rely upon an ongoing and systematic data infrastructure of housing conditions that is foundational for policy and research, such as the English Housing Survey (EHS) (www.gov.uk/government/statistics/english-housing-survey-2021-to-2022-headline-report) and the American Housing Survey (AHS) (https://census.gov/programs-surveys/ahs.html). This project fits within a larger series of linked housing conditions data infrastructures, collected in 2016^4,5^, 2020^6,7^, and now most recently in 2022^8^. The data infrastructures have been widely used by researchers^9^, government^10^, and non-government organisations^11^, to describe and analyse, for example, the prevalence of cold housing^12^, model energy use^13^, or to estimate the impact of housing on mental health during COVID-19^14^.

The data infrastructure series directly responded to an acknowledged lack of up-to-date, reliable, and accessible data on housing conditions in Australia. The first project in this series, titled the Australian Housing Conditions Dataset (2016 AHCD) responded to the need to create a housing conditions data infrastructure in Australia almost two decades after the last Australian Housing Survey (1999) (https://www.abs.gov.au/ausstats/abs@.nsf/dossbytitle/949017CAABBD0B6ECA256BD00027B1CB?OpenDocument) had been undertaken by the Australian Bureau of Statistics. The second project in the series was the Australian Rental Housing Conditions Dataset (2020 ARHCD). The ARHCD focused solely on housing conditions in privately and publicly rented properties. This most recent data infrastructure extends across the major tenures to include households in home ownership, home purchase, and rental.

Since World War II, home ownership has been the dominant tenure in Australia. Yet it is now in decline – a trend that is also evident in all other market liberal countries. Australian Bureau of Statistics Census data reveals a long-run and significant decrease in home ownership, especially among young people^15^. The decline of home ownership can be attributed to various factors, including a changing labour market, decreased affordability, and the expansion of the rental market^16^. Renting is now the fastest growing tenure in Australia, with more than one third of Australians renting their homes, either publicly or privately^17^. Although it was once expected that, for most, renting was a transitionary tenure that preceded home ownership, recent research has shown that Australians are now more likely than ever to be long-term renters or even rent for their whole adult lives^18^.

This large-scale national project was enabled by funding from the Australian Research Council (ARC) through the Linkage Infrastructure, Equipment and Facilities (LIEF) grant program in partnership with the University of Adelaide, the University of South Australia, the University of Melbourne, Swinburne University of Technology, Curtin University, and Torrens University Australia.

## Methods

This data infrastructure captures 22,550 Australian households. Survey data were collected between August and October 2022.

The research team devised the project and designed the data collection tool. Funding was granted by the Australian Research Council under a Linkage, Infrastructure, Equipment and Facilities scheme, and ethical approval was permitted by the University of Adelaide’s Human Research Ethics Committee (H-2020-069). A market research agency was commissioned to support with the survey development, data collection, data cleaning and testing. Upon receiving the cleaned dataset, the research team liaised with the Australian Data Archive (ADA) to organise data lodgement into the repository and ongoing management of the data.

### Questionnaire development

Most of the survey questions were drawn from the former two surveys in the series of Australian Housing Conditions projects. This has ensured that researchers can analyse the datasets longitudinally (acknowledging that the survey series is repeat cross-sectional as opposed to a true longitudinal panel sample). These questionnaires were developed by the investigators.

To enable comparison across jurisdictions and time, several new crossover questions were drawn from a variety of continuing and long-standing international housing and household surveys, including the English Housing Survey and the Australian Housing and Urban Research Institute’s Australian Housing Aspirations (AHA) Survey^19^. Other relevant international surveys were reviewed for relevance, including the American Housing Survey and the English Private Landlord Survey (https://www.gov.uk/government/statistics/english-private-landlord-survey-2021-main-report). The research team also designed some entirely new questions for the survey. These questions were intentionally designed to capture pressing and emerging policy issues in housing, especially regarding the changing role of homeownership in Australia. Some examples include whether renters plan to buy property, whether renters or homeowners own multiple properties, and for what reasons these participants own additional properties.

The survey was organised into three parts. Following the respondent screening questions at the outset of the questionnaire, the first part asked participants to answer basic questions regarding their housing profile, i.e., whether they rent or own their home, the type of dwelling they inhabit, and the length of their tenure. Participants were also asked to share details about their home’s conditions pertaining to heating and cooling, affordability, security, and satisfaction with the dwelling quality. This section ended with a focus on participants’ future housing intentions. The second section focussed on housing and its impact on health, including whether participants had ever been injured due to their dwelling being unsafe or of poor quality. The final section of the survey posed questions relating to household demographics and finances.

### Sampling

All participants were required to be over the age of 18 to participate in the survey. The survey was provided in English. Data collection quotas were applied so that the data is reflective of the distribution of households across Australian states and territories. Quotas were also applied to replicate tenure and rental type distributions. In recognition of the emerging importance of rental tenure and the call for detailed work on this tenure, the sampling frame was weighted to oversample rental households. This variation is illustrated in Table 1, with the highest number of private rental, social rental, and homeowner responses being recorded in New South Wales as a result of the state’s higher population compared to other Australian jurisdictions.

**Table 1:** Composition of the final dataset by jurisdiction and tenancy type.

	Total	NSW	VIC	QLD	SA	WA	TAS	NT	ACT
**Households**	**22,550**	**7,151**	**5,546**	**4,808**	**1,670**	**2,272**	**505**	**198**	**400**
**Total %**	**100%**	**31.7%**	**24.6%**	**21.3%**	**7.4%**	**10.1%**	**2.2%**	**0.09%**	**1.8%**
Private rental	12,930	4,139	3,171	2,976	816	1229	245	141	213
Social rental	2,052	675	390	382	253	211	70	13	58
Homeowners	7,568	2,337	1,985	1,450	601	832	190	44	129

Participants were recruited through online and offline methods to minimise technology affinity bias. Offline methods include telephone or face to face recruitment. Online methods include recruitment through social networks, loyalty websites, affiliate traffic, and the panel’s own newsletters. The market research agency utilised a representative panel that is targeted to align with Australian Bureau of Statistics (ABS) Census data (see Supplementary Table 1). As a result, the sampling was designed to be broadly representative of the Australian population.

### Data collection

Two survey pilots (n = 104 and n = 100) were undertaken to test scripting logic within the survey. The full data collection commenced 26 August 2022 and concluded 18 October 2022. The survey took approximately 10 minutes for participants to complete.

### Composition of the final dataset

The final dataset comprises responses from 22,550 Australian households. Altogether the renters in social housing (n = 2,052), private renters (n = 12,930) and homeowners (n = 7,568) quotas were successfully met. Table 1 shows the final sample by location (state or territory) and tenancy type (homeowner, social, or private rental). In accordance with the ethics approval, all respondents gave their informed consent at the start of the survey. Their consent was attained under the conditions that the data were de-identified prior to analysis or sharing, that the data were securely stored, and that the data were to be used for research and policy purposes only.

## Data Records

The 2022 AHCD is lodged with the Australian Data Archive (reference number 100133)^8^. Both sensitive and non-sensitive versions are accessible to researchers and the public via https://dataverse.ada.edu.au/dataset.xhtml?persistentId=doi:10.26193/SLCU9J upon registration and request. The data are as received from the commissioned agency except for minor changes.

The sensitive portion of the dataset is under separate access conditions at the data repository due to its inclusion of sensitive variables such as postcodes and annual income. This is consistent with the repository’s efforts to protect participants’ personal data and limit re-identification. These measures also ensure that the research team can maintain a record of the researchers who request access to these sensitive portions of the data.

Some parts of the non-sensitive data have been aggregated to remove the sensitive elements (such as the annual income variable), and some questions have been removed (such as the postcodes). While the non-sensitive dataset poses a lower risk of re-identification of participants due to these modifications, users still must register to the data repository to gain access to the dataset, albeit these are lighter controls relative to the sensitive dataset. Registration to the repository is required by our ethical committee to ensure that all researchers have read and agreed to the conditions of use.

The only other manipulation to the dataset that has occurred is the shortening of labels to avoid truncation in STATA. Both versions of the dataset contain all survey responses except for open-ended questions and responses to “Other (please type in)”. These responses are only accessible to the research team to protect participant privacy and prevent any possible reidentification of data. The details of the two versions (sensitive and non-sensitive) of the AHCD are provided in Table 2. The AHCD files accessible via the ADA are available in.csv,.sas,.sav, and.dta formats.

**Table 2:** File names and types as stored on Australian Data Archive.

File name	File type	Notes
2_HousingConditions_2022_CSV_100133_GENERAL.zip	.csv;.sas;.sav;.dta	Final *non-sensitive* version of the dataset without postcode variable and with an aggregated annual income variable. All survey responses except for open-ended questions or responses to “Other, (please type in)”. Access upon registration and request, managed by ADA.
2_HousingConditions_2022_SAS_100133_GENERAL.zip		
2_HousingConditions_2022_SPSS_100133_GENERAL.zip		
2_HousingConditions_2022_STATA_100133_GENERAL.zip		
3_HousingConditions_2022_CSV_100133_RESTRICTED.zip	.csv;.sas;.sav;.dta	Final *sensitive* version of the dataset containing the postcode variable and original annual income variable. All survey responses except for open-ended questions or responses to “Other, (please type in)”. Access upon registration and request, managed by ADA.
3_HousingConditions_2022_SAS_100133_ RESTRICTED.zip		
3_HousingConditions_2022_SPSS_100133_ RESTRICTED.zip		
3_HousingConditions_2022_STATA_100133_ RESTRICTED zip		

## Technical Validation

### Data checking and cleaning protocols

All data processing requirements were conducted in-house by the commercial provider. While potential issues with the data were addressed via logic checks at the programming stage (e.g., restricting the types of data which can entered), a range of data checks were also completed upon commencement of fieldwork, including after the pilot survey. This process involved the following data cleaning and validation activities:

- Confirmation that the survey logic was working correctly;
- Validation that data were captured as per the required format and with only permissible values (e.g., that the correct question format was adopted such as multiple versus single responses);
- Verification of correct labelling of variables and values;
- Verification of any inconsistent response categories; and,
- De-identification of data to protect the privacy and confidentiality of individuals who participated in the survey.

Following fieldwork, data were exported to Q and SPSS, which was reviewed and validated by the agency’s project team members. They performed a final check of survey logic, and verbatim responses were cleaned of any identifying information, including names and contact details of individuals before draft and final datasets and files were provided to the University.

Subsequent testing of data quality was undertaken between December and April by the investigators.

The market research firm undertook a variety of measures to ensure that participants’ privacy was safeguarded. These include:

- Only collecting personal information required as part of the research;
- Not disclosing any personally identifiable information that was collected, unless with the participants’ prior consent to do so;
- Providing participants with the firm’s privacy policy; and,
- Complying with a range of data storage and privacy protocols, frameworks, and international standards.

## Usage Notes

To maximise the utility of the AHCD for housing and urban research, users may consider adding functionality to the dataset by geo-coding responses (postcodes available in the sensitive version only), or by formulating design and/or non-response weights.

The sensitive human data contained in this dataset is available for any user who reads and agrees to the Data Usage terms at ADA. These are required to protect personal data from participants. Users of the dataset must agree to these conditions:

- Do not re-distribute the data (the agreement is for a single person, every collaborator needs to apply);
- Do not sell the data;
- Do not attempt to identify any participant; and,
- Do not attempt to contact any participant for any reason.

### Supplementary information

Supplementary Information

## Data Availability

No custom code was used.
