# Supplementary material for: An Australian housing conditions data infrastructure
Source: Sci Data. 2023 Nov 21;10:817. doi: 10.1038/s41597-023-02739-2 (PMC10663474; doi:10.1038/s41597-023-02739-2)
Supplement: Supplementary file 1 — Supplementary Information [file 41597_2023_2739_MOESM1_ESM.docx]

Supplementary Table 1: Comparison of the sex, age, household income, dwelling type and landlord type of the Australian Housing Conditions Dataset (AHCD) homeowner and renter samples with the 2021 ABS Census*, by State or Territory

| Variable | Category (source) | **Australian State or Territory** | | | | | | | | | | | | | | | | | | |
| --- | --- | --- | --- | --- | --- | --- | --- | --- | --- | --- | --- | --- | --- | --- | --- | --- | --- | --- | --- | --- |
|  |  | **NSW** | | **VIC** | | **QLD** | | **SA** | | **WA** | | **TAS** | | **NT** | | **ACT** | | **ALL** | | |
|  |  | Own | Rent | Own | Rent | Own | Rent | Own | Rent | Own | Rent | Own | Rent | Own | Rent | Own | Rent | Own | Rent |  |
| **Sex / Gender** | Male (AHCD) | 33.2 | 23.2 | 30.4 | 22.9 | 27.1 | 21.3 | 34.3 | 27.3 | 30.5 | 24.0 | 26.8 | 24.4 | 31.8 | 22.1 | 25.6 | 21.0 | 30.8 | 23.1 |  |
|  | Male (2021 Census) | 48.3 | 49.3 | 48.2 | 49.3 | 48.2 | 47.9 | 48.6 | 48.8 | 48.1 | 48.4 | 48.4 | 48.1 | 48.9 | 48.4 | 48.1 | 49.7 | 48.3 | 48.9 |  |
|  | Female (AHCD) | 66.3 | 75.8 | 69.2 | 76.1 | 72.2 | 77.6 | 65.4 | 71.8 | 69.4 | 75.1 | 73.2 | 74.3 | 68.2 | 77.3 | 74.4 | 78.2 | 68.8 | 76.0 |  |
|  | Female (2021 Census) | 51.7 | 50.7 | 51.8 | 50.7 | 51.8 | 52.1 | 51.4 | 51.2 | 51.9 | 51.6 | 51.6 | 51.9 | 51.1 | 51.6 | 51.9 | 50.3 | 51.7 | 51.1 |  |
|  | | | | | | | | | | | | | | | | | | | | |
| **Age** | 18 to 29 years (AHCD) | 27.6 | 45.6 | 29.2 | 46.1 | 26.1 | 45.0 | 18.0 | 37.0 | 16.5 | 43.3 | 17.4 | 40.3 | 13.6 | 45.5 | 20.2 | 49.4 | 25.3 | 44.7 |  |
|  | 18 to 29 years (2021 Census) | 14.7 | 29.7 | 15.3 | 32.9 | 13.6 | 31.8 | 14.7 | 30.8 | 14.9 | 30.6 | 13.4 | 31.8 | 13.3 | 30.9 | 15.6 | 38.4 | 14.7 | 31.3 |  |
|  | 30 to 49 years (AHCD) | 38.9 | 39.3 | 39.0 | 40.4 | 32.3 | 36.0 | 36.9 | 38.3 | 43.0 | 38.5 | 38.4 | 41.0 | 47.7 | 37.7 | 48.1 | 36.9 | 38.1 | 38.7 |  |
|  | 30 to 49 years (2021 Census) | 31.8 | 43.5 | 33.9 | 43.7 | 32.3 | 40.1 | 30.5 | 39.4 | 34.4 | 42.3 | 28.6 | 39.3 | 35.6 | 43.5 | 38.3 | 43.0 | 32.7 | 42.4 |  |
|  | 50 to 64 years (AHCD) | 15.9 | 10.0 | 15.3 | 9.1 | 19.1 | 12.2 | 26.6 | 16.6 | 24.8 | 11.2 | 26.3 | 14.0 | 18.2 | 10.4 | 19.4 | 10.3 | 18.5 | 10.9 |  |
|  | 50 to 64 years (2021 Census) | 26.8 | 16.9 | 26.2 | 15.1 | 27.4 | 17.7 | 27.3 | 17.8 | 26.7 | 17.0 | 28.6 | 16.7 | 29.5 | 17.5 | 24.8 | 12.4 | 26.8 | 16.6 |  |
|  | 65 years or over (AHCD) | 17.4 | 4.8 | 16.4 | 4.3 | 22.0 | 6.8 | 18.5 | 8.1 | 15.6 | 6.9 | 17.9 | 4.8 | 20.5 | 5.8 | 12.4 | 3.3 | 17.9 | 5.5 |  |
|  | 65 years or over (2021 Census) | 26.7 | 9.8 | 24.6 | 8.3 | 26.7 | 10.5 | 27.5 | 12.0 | 24.0 | 10.1 | 29.4 | 12.1 | 21.7 | 8.1 | 21.2 | 6.2 | 25.8 | 9.7 |  |
|  | | | | | | | | | | | | | | | | | | | | |
| **Household Income** | Under $31,000 (AHCD) | 5.9 | 11.9 | 6.6 | 12.8 | 6.6 | 12.7 | 5.8 | 18.4 | 4.2 | 13.7 | 4.2 | 26.3 | 0.0 | 7.1 | 0.8 | 13.3 | 5.9 | 13.2 |  |
|  | Less Than $33,800 (2021 Census) | 8.5 | 11.7 | 9.0 | 11.7 | 8.9 | 11.3 | 9.5 | 16.7 | 8.4 | 12.6 | 10.8 | 16.9 | 5.7 | 9.2 | 3.7 | 8.0 | 8.7 | 12.1 |  |
|  | $31,000 - $59,000 (AHCD) | 10.3 | 15.6 | 11.4 | 16.4 | 15.8 | 17.4 | 15.8 | 23.3 | 11.5 | 14.6 | 15.3 | 21.0 | 9.1 | 7.1 | 4.7 | 12.5 | 12.2 | 16.6 |  |
|  | $33,800 - $64,999 (2021 Census) | 14.3 | 16.4 | 15.0 | 17.1 | 16.0 | 19.0 | 18.3 | 21.8 | 14.1 | 17.3 | 20.4 | 22.9 | 10.4 | 15.5 | 7.9 | 10.6 | 15.1 | 17.6 |  |
|  | $59,001 to $90,000 (AHCD) | 15.1 | 19.7 | 15.8 | 20.5 | 15.4 | 20.6 | 19.3 | 18.4 | 13.9 | 20.8 | 24.7 | 21.3 | 18.2 | 13.0 | 12.4 | 15.1 | 15.8 | 20.0 |  |
|  | $65,000 - $90,999 (2021 Census) | 10.5 | 13.9 | 11.5 | 15.0 | 11.3 | 16.2 | 12.9 | 16.0 | 10.5 | 14.2 | 13.7 | 15.9 | 8.9 | 13.8 | 8.8 | 12.0 | 11.1 | 14.8 |  |
|  | $90,001 to $125,000 (AHCD) | 14.1 | 15.4 | 17.0 | 16.3 | 15.5 | 16.0 | 17.3 | 14.7 | 18.0 | 13.9 | 17.9 | 12.4 | 15.9 | 18.2 | 21.7 | 15.5 | 16.1 | 15.5 |  |
|  | $91,000 - $129,999 (2021 Census) | 15.8 | 19.3 | 17.2 | 19.9 | 17.0 | 20.7 | 17.9 | 18.3 | 16.5 | 18.7 | 17.9 | 17.5 | 16.1 | 18.8 | 15.6 | 20.3 | 16.7 | 19.6 |  |
|  | $125,001 to $150,000 (AHCD) | 12.6 | 10.2 | 13.0 | 9.5 | 13.1 | 10.4 | 9.5 | 6.7 | 12.1 | 9.9 | 9.5 | 6.3 | 4.5 | 16.9 | 14.0 | 8.5 | 12.4 | 9.7 |  |
|  | $130,000 - $155,999 (2021 Census) | 8.7 | 8.5 | 9.2 | 8.7 | 9.2 | 8.5 | 9.4 | 7.1 | 9.1 | 7.8 | 8.8 | 6.4 | 9.3 | 8.6 | 8.6 | 10.1 | 9.0 | 8.4 |  |
|  | $150,001 to $175,000 (AHCD) | 7.5 | 6.5 | 7.9 | 6.3 | 7.9 | 5.6 | 8.0 | 3.6 | 8.7 | 5.8 | 2.6 | 2.9 | 11.4 | 5.2 | 6.2 | 10.0 | 7.7 | 6.0 |  |
|  | $156,000 - $181,999 (2021 Census) | 8.1 | 7.0 | 8.2 | 6.7 | 8.1 | 6.0 | 7.6 | 4.8 | 8.5 | 6.4 | 6.9 | 4.4 | 9.6 | 6.6 | 9.6 | 8.9 | 8.1 | 6.5 |  |
|  | $175,001 to $200,000 (AHCD) | 8.3 | 5.0 | 7.7 | 4.2 | 6.8 | 4.4 | 5.7 | 2.6 | 9.0 | 5.3 | 8.4 | 2.9 | 9.1 | 7.1 | 10.1 | 4.4 | 7.7 | 4.5 |  |
|  | $182,000 - $207,999 (2021 Census) | 5.9 | 4.5 | 5.7 | 4.2 | 5.7 | 3.5 | 5.1 | 2.7 | 5.7 | 3.8 | 4.5 | 2.7 | 7.3 | 4.6 | 8.3 | 6.2 | 5.7 | 4.0 |  |
|  | Over $200,000 (AHCD) | 15.2 | 7.3 | 11.3 | 5.7 | 10.4 | 5.1 | 8.7 | 4.1 | 12.0 | 6.8 | 6.8 | 2.2 | 13.6 | 11.0 | 19.4 | 14.4 | 12.2 | 6.2 |  |
|  | Over $208,000 (2021 Census) | 22.6 | 12.8 | 18.7 | 10.7 | 17.2 | 7.8 | 13.5 | 5.6 | 19.3 | 10.6 | 10.5 | 5.2 | 23.3 | 10.6 | 33.2 | 16.7 | 19.4 | 10.5 |  |
|  | | | | | | | | | | | | | | | | | | | | |
| **Dwelling Type** | Separate house (AHCD) | 73.9 | 41.0 | 78.7 | 49.6 | 79.8 | 51.1 | 86.0 | 60.5 | 85.9 | 56.6 | 93.7 | 66.0 | 70.5 | 39.0 | 61.2 | 38.7 | 78.8 | 48.7 |  |
|  | Separate house (2021 Census) | 78.3 | 41.4 | 83.0 | 51.3 | 84.9 | 54.5 | 87.5 | 56.7 | 86.9 | 60.5 | 94.8 | 70.0 | 72.5 | 50.6 | 74.3 | 39.9 | 82.8 | 50.1 |  |
|  | Semi-detached, row or terrace house, or townhouse (AHCD) | 10.6 | 17.3 | 11.0 | 20.2 | 9.9 | 20.4 | 9.7 | 23.6 | 8.3 | 19.2 | 3.7 | 16.5 | 13.6 | 13.6 | 20.2 | 25.1 | 10.2 | 19.4 |  |
|  | Semi-detached, row or terrace house, or townhouse (2021 Census) | 9.3 | 15.9 | 10.5 | 21.2 | 6.8 | 19.8 | 8.9 | 25.9 | 8.4 | 22.7 | 2.5 | 14.7 | 6.5 | 17.2 | 13.9 | 23.3 | 8.9 | 19.4 |  |
|  | Flat or apartment with 4 or less floors (AHCD) | 9.3 | 25.8 | 5.2 | 17.7 | 5.8 | 16.7 | 2.5 | 12.8 | 3.5 | 15.8 | 1.6 | 12.1 | 6.8 | 25.3 | 10.9 | 21.8 | 6.2 | 19.6 |  |
|  | Flat or apartment with 4 or less floors (2021 Census) | 5.2 | 19.9 | 3.3 | 13.3 | 3.1 | 14.2 | 2.0 | 13.2 | 1.5 | 8.9 | 1.7 | 12.8 | 4.7 | 13.5 | 4.4 | 15.7 | 3.5 | 15.3 |  |
|  | Flat or apartment with more than 4 floors (AHCD) | 5.2 | 14.0 | 3.6 | 9.9 | 3.0 | 9.3 | 1.3 | 1.5 | 1.1 | 5.6 | 0.0 | 1.6 | 6.8 | 16.2 | 7.8 | 14.0 | 3.5 | 10.0 |  |
|  | Flat or apartment with more than 4 floors (2021 Census) | 6.2 | 20.7 | 2.7 | 13.2 | 3.2 | 9.5 | 0.7 | 2.9 | 1.4 | 5.9 | 0.1 | 0.8 | 3.9 | 12.6 | 7.1 | 20.1 | 3.6 | 13.4 |  |
|  | Other (AHCD) | 0.9 | 1.9 | 1.5 | 2.5 | 1.4 | 2.4 | 0.5 | 1.6 | 1.2 | 2.8 | 1.1 | 3.8 | 2.3 | 5.8 | 0.0 | 0.4 | 1.2 | 2.3 |  |
|  | Other (2021 Census) | 0.8 | 1.6 | 0.4 | 0.8 | 1.7 | 1.3 | 0.6 | 0.8 | 1.6 | 1.6 | 0.6 | 1.4 | 10.7 | 4.0 | 0.2 | 0.6 | 1.0 | 1.3 |  |
|  | | | | | | | | | | | | | | | | | | | | |
| **Landlord type** | A real estate agent (AHCD) |  | 69.7 |  | 74.2 |  | 67.9 |  | 52.9 |  | 55.8 |  | 49.5 |  | 63.6 |  | 57.6 |  | 67.1 |  |
|  | A real estate agent (2021 Census) |  | 70.6 |  | 73.3 |  | 66.8 |  | 51.9 |  | 54.9 |  | 47.8 |  | 36.2 |  | 56.2 |  | 14.8 |  |
|  | A State or Territory housing authority (AHCD) |  | 6.1 |  | 5.3 |  | 6.0 |  | 13.9 |  | 8.1 |  | 11.4 |  | 5.8 |  | 13.3 |  | 6.9 |  |
|  | A State or Territory housing authority (2021 Census) |  | 8.2 |  | 6.2 |  | 7.1 |  | 13.2 |  | 9.5 |  | 12.8 |  | 26.0 |  | 13.6 |  | 28.9 |  |
|  | Someone not in the same household (AHCD) |  | 11.2 |  | 9.3 |  | 12.7 |  | 16.6 |  | 19.0 |  | 23.2 |  | 11.7 |  | 13.3 |  | 12.5 |  |
|  | Someone not in the same household (2021 Census) |  | 14.9 |  | 15.7 |  | 17.5 |  | 25.0 |  | 25.0 |  | 29.5 |  | 14.2 |  | 22.1 |  | 17.6 |  |
|  | A community housing provider (AHCD) |  | 7.9 |  | 5.6 |  | 5.3 |  | 9.7 |  | 6.5 |  | 10.8 |  | 2.6 |  | 8.1 |  | 6.8 |  |
|  | A community housing provider (2021 Census) |  | 2.4 |  | 1.4 |  | 1.6 |  | 4.0 |  | 2.5 |  | 3.9 |  | 9.8 |  | 1.7 |  | 2.2 |  |
|  | Other (AHCD) |  | 5.1 |  | 5.5 |  | 8.0 |  | 6.8 |  | 10.5 |  | 5.1 |  | 16.2 |  | 7.7 |  | 6.6 |  |
|  | Other (2021 Census) |  | 3.3 |  | 2.9 |  | 6.4 |  | 5.1 |  | 7.5 |  | 5.1 |  | 12.9 |  | 6.0 |  | 4.6 |  |

*2021 Census Data is based on place of enumeration and is using the count of persons for all data, except dwelling type which is counted by dwellings.
